# Differential Role of Poly(ADP-ribose) polymerase in *D. discoideum *growth and development

**DOI:** 10.1186/1471-213X-11-14

**Published:** 2011-03-09

**Authors:** Jyotika Rajawat, Hina Mir, Rasheedunnisa Begum

**Affiliations:** 1 Department of Biochemistry, Faculty of Science, The Maharaja Sayajirao University of Baroda, Vadodara-390002, Gujarat, India

## Abstract

**Background:**

Poly(ADP-ribose) polymerase is evolutionarily conserved as a responder to various forms of stress. Though PARP's role in cell death is well addressed, its role in development and multicellularity is still an enigma. We have previously reported the role of PARP in oxidative stress induced delayed development of *D. discoideum*.

**Results:**

In the current study we highlight the involvement of PARP during *D. discoideum *development. Oxidative stress affects expression of *aca *and *cAR*1 thus affecting aggregation. Although *parp *expression is not affected during oxidative stress but it is involved during normal development as confirmed by our PARP down-regulation studies. Constitutive PARP down-regulation resulted in blocked development while no effect was observed on *D. discoideum *growth. Interestingly, stage specific PARP down-regulation arrested development at the slug stage.

**Conclusion:**

These results emphasize that PARP is essential for complex differentiation and its function may be linked to multicellularity. This is the first report where the involvement of PARP during normal multicellular development in *D. discoideum*, an ancient eukaryote, is established which could be of evolutionary significance. Thus our study adds one more role to the multitasking function of PARP.

## Background

Poly(ADP-ribose) polymerase-1 (PARP-1) protects the genome by functioning in the DNA damage repair network. Gene disruption studies involving PARP have identified various roles of PARP in cellular responses to DNA damage. *Parp*-/- mice are resistant to DNA damage induced cell death suggesting the involvement of PARP in cell death through NAD^+ ^depletion [1]. PARP is a DNA damage sensor which upon binding to damaged sites triggers transfer of long, linear or branched chains of poly(ADP-ribose) (PAR) onto various nuclear acceptor proteins, including itself at the expense of NAD^+ ^[2]. PARP is also a mediator of cell death after ischemia, reperfusion injury and exposure to various DNA damaging agents [3]. PARP has been shown to promote caspase independent cell death *via *release of apoptosis inducing factor [4,5]. Recently PARP's role has been identified in cytochrome c release during NMDA induced excitotoxicity [6]. The oxidant- and free-radical mediated necrosis of pancreatic β-cells, neurons, thymocytes and other cell types can be prevented by PARP inhibitors [7].

Interesting observation that cells do exhibit alternate pathways to undergo cell death which are caspase independent has evoked interest in PARP and its role in these alternate pathways of cell death. Much interest has been emerging to understand the precise mechanism by which PARP mediates genome stabilization and protection against damage, as well as its involvement in different types of cell death. To study the function of PARP, PARP inhibitors are extensively used. However, these inhibitors have been reported to have nonspecific effects on other metabolites. This led us to the use of molecular biology approaches for modulation of poly ADP-ribosylation in living cells. We have studied the dose dependent effect of hydroxylamine (*in situ *H_2_O_2 _generation) on *D. discoideum *development and also the role of PARP during oxidative stress induced effects on development [8]. In the current study PARP expression is down-regulated using antisense approach and we have established for the first time *D. discoideum *as a model for the constitutive as well as stage specific inducible antisense of PARP.

Our PARP inhibition results with benzamide suggest that under oxidative stress PARP gets activated within 5-10 minutes. The developmental defect seen at 10 mM benzamide could be either due to strong inhibition as observed by PARP activity (data not shown) or due to nonspecific effect, benzamide being a nicotinamide analogue. To support these results and to rule out any non specific effect of benzamide, we made an attempt in this study to specifically down-regulate PARP by antisense and check its effect on oxidative stress induced development. *D. discoideum *possesses more than one type of PARP [9] nevertheless their catalytic domains are highly conserved, hence, the catalytic domain was used as a target for down-regulation.

## Methods

### *D. discoideum *culturing conditions

*D. discoideum *Ax-2 strain which is an axenic derivative of Raper's wild type NC-4 (a mutant in at least two genes i.e. *axe *A and *axe *B) was used. *D. discoideum *was grown under different culture conditions. The growing cells (unicellular) were maintained in a liquid suspension (HL5 medium). *D. discoideum *cells were grown in HL5 medium, pH 6.5 with 150 rpm shaking at 22°C [10]. Log phase cells at a density of ~2.5 × 10^6 ^cells/ml were used for experiments.

*D. discoideum *was maintained on a solid substratum containing Phosphate Buffered Agar (PBA). *D. discoideum *was also cultured on bacterial lawn of *Klebsiella *which is its natural food. For this a loop full of overnight grown culture of *Klebsiella *was taken and *D. discoideum *spores (4-5) were mixed with it. This 'mixture' of two cell types is then pour plated on PBA plates (90 mm). *D. discoideum *cells fed on *Klebsiella *and when no more *Klebsiella *was left the cells undergo developmental changes and form fruiting bodies.

### Monitoring growth of *D. discoideum *cells

1.5 × 10^6 ^cells were harvested, washed with 1X Sorenson's buffer (SB) and finally resuspended in 4 ml of sterile HL5 containing flask and growth was monitored for 6 days at an interval of 12 hours under shaking conditions at 22°C.

### Induction of oxidative stress

Oxidative stress was induced in *D. discoideum *cells by *in situ *generation of H_2_O_2 _upon addition of hydroxylamine (HA) (Sigma), [11]. Log phase cells at a density of ~2.5 × 10^6 ^cells/ml were exposed to different doses of HA (0, 1, 2.5, 4 mM) in HL5 medium at 22°C in a sterile flask.

### Effect of benzamide on the development of *D. discoideum *cells

1.5 × 10^6 ^cells were treated with the benzamide (0, 1, 2, 3, 4, 6, 10 mM) for 12 hours. Following this, the cells (pre-exposed to benzamide) were then washed with 1X SB and the pellet was resuspended in 100 μl of 1X SB and spread on PBA plate [12]. Different developmental stages were monitored at an interval of 6 hours.

### Expression analysis of *aca, cAR*1, *yak*A, *parp, countin*50, *gp*80 *and hsp*D by RT-PCR

*D. discoideum *cells were exposed to oxidative stress as mentioned earlier. After one hour pretreatment cells were pelleted and washed with 1X SB and finally resuspended in 1X SB. Total RNA was isolated from the cells at two time points (6 and 10 hours) using TRIZOL reagent (Invitrogen, USA). The expression kinetics of *aca*A, *cAR*1, *yak*A, *parp*, *countin*50, *gp*80 and *hsp*D was examined by RT-PCR and *rnl*A was used as an internal control. The reactions were performed according to the manufacturer's instructions (Fermentas, Ontario, Canada). DNA fragments were amplified for 24 cycles after reverse transcription and signal intensities were analyzed on 2% agarose gel.

### Strategy for targeted down-regulation of *adprt*1A encoding PARP

Antisense of 500 bp was designed for the catalytic domain of PARP. This region was PCR amplified using oligonucleotide primers (left primer: **5'**AAAACGGGTTCCTCACTTTG**3' **and right primer: **5'**CGGCGATTAGAATTCTTCGT **3'**). The confirmed amplified product was cloned in Bluescript KS+ vector. Randomly selected white colonies were screened for the presence of recombinant plasmid which was confirmed by restriction digestion pattern. This recombinant plasmid was used as an intermediate plasmid for further cloning in the target vectors (constitutive and stage specific). For cloning in pTX vector, the intermediate recombinant plasmid was digested with *KpnI *and *BamHI *and ligated with *KpnI *and *BamHI *digested pTX. Presence of PARP antisense insert was confirmed by colony PCR and relevant restriction enzyme digestion patterns. The confirmed clone containing PARP antisense, pTX-PARP, was used for transformation of *D. discoideum *cells.

Similar strategy was also followed for cloning PARP antisense in a stage specific vector EcmB using *SmaI *and *XhoI *enzymes. Clones obtained were screened by relevant restriction enzyme digestion and confirmed by PCR and was named as EcmB-PARP. The confirmed clones i.e., pTX-PARP and EcmB-PARP were independently used to generate *D. discoideum *transformants with constitutive and inducible down-regulation of PARP respectively.

### Measurement of PARP activity by indirect immunofluorescence [13]

PARP activity was assayed by using antibodies against the product of PARP i.e. PAR. For assaying PARP, indirect immunofluorescence was done using anti-PAR mouse mAb (10 H) (Calbiochem, Germany) at a concentration of 0.5 μg/ml and anti-mouse IgG (secondary antibody)-FITC conjugate (Sigma) at a dilution of 1:200. *D. discoideum *cells were pelleted and washed once with phosphate buffered saline (PBS) pH 7.4, fixed in 70% chilled methanol for 10 minutes at -20°C and then washed with blocking solution (1.5% BSA with 0.05% Tween 20 in PBS) followed by incubation for 1 hour in primary antibody. After incubation the cells were washed 2-3 times with blocking solution and further incubated for 1 hour with FITC labeled secondary antibody. Finally these cells were washed 2-3 times with PBS and the fluorescence was observed at 490 nm under 60X magnification.

## Results

### *D. discoideum *development under oxidative stress

**Figure 1:**
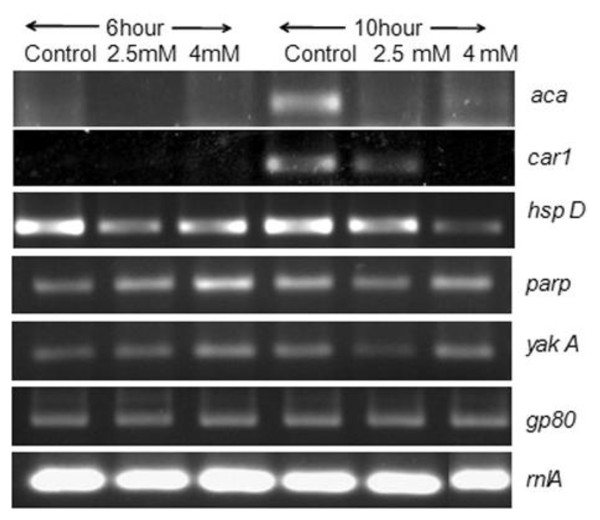
**Expression profile of genes controlling early development in *D. discoideum***. Expression of cAMP receptor- *cAR*1 and adenylyl cyclase A- *aca*A were found to be decreased in HA treated cells. Expression of other genes was unchanged. *rnl*A (mitochondrial rRNA IG7) was used as an internal control.

*D. discoideum *developmental studies were performed to explore the effect of oxidative stress on development and differentiation. Our previous report [8] suggests that the most significant effect of oxidative stress seemed to occur at loose aggregation stage. Therefore the expression of genes involved during aggregation was analyzed after treatment with 2.5 and 4 mM HA (LD_50 _and LD_90 _respectively). mRNA levels of *yak*A and *car*1 were assayed at 0 hour also while rest of the genes were assayed at 6 and 10 hours of development induction. The developmental expression pattern of *aca*A, *cAR*1, *yak*A, *parp*, *countin*50, *gp*80 and *hsp*D are shown in Figure 1. As judged by RT-PCR analysis, expression of *adenylyl cyclase *A *(aca*A) and cAMP receptor 1 (*car*1) was affected during oxidative stress. Expression of *aca*A at 6 hours and *car*1 at 10 hours was found to decrease in a dose dependent manner at 2.5 and 4 mM HA doses, while expression of other genes was comparable to control. Also there was no change in the expression of these genes in presence of benzamide (data not shown).

### Role of PARP during *D. discoideum *development

**Figure 2:**
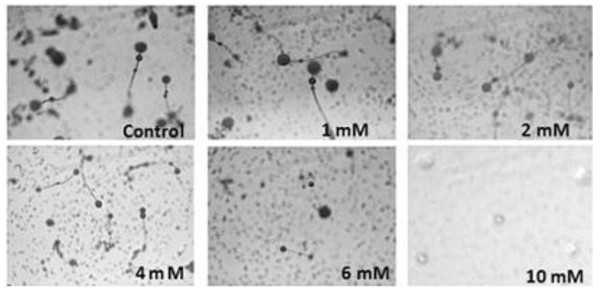
**Monitoring development in *D. discoideum *cells during PARP inhibition by benzamide**. The development was monitored after 24 hours. The photographs were taken under 4X objective.

The role of PARP in *D. discoideum *development was investigated by its inhibition with benzamide. 1.0, 2.0 and 3.0 mM benzamide did not show any effect on development. However, benzamide at 4 mM dose delayed the transition from tight aggregate (TA) to slug by 3-4 hours (Table 1). Intriguingly 10 mM benzamide arrested the development at loose aggregation stage (Figure 2). This suggests a plausible role of PARP during development. Interestingly, 4.0 and 6.0 mM benzamide treated *D. discoideum *cells showed abnormal fruiting bodies with bigger size fruits (Figure 2).

**Table 1:** Effect of PARP inhibitor, benzamide on *D. discoideum *development

Benz (mM)	LA (hr)	TA (hr)	SF (hr)	FBF (hr)	% CD	% FB
0.0	**6**	**12**	**18**	**24**	**1**	100

1.0	6	12	18	24	2	100

2.0	6	12	18	24	2	100

3.0	6	12	18	24	4	95

4.0	6	12	22	28	10	95

LA: Loose aggregate; TA: Tight aggregate; SF: Slug formation; FBF: Fruiting body formation, CD: cell death; FB: Fruiting body

### Functional characterization of PARP antisense

**Figure 3:**
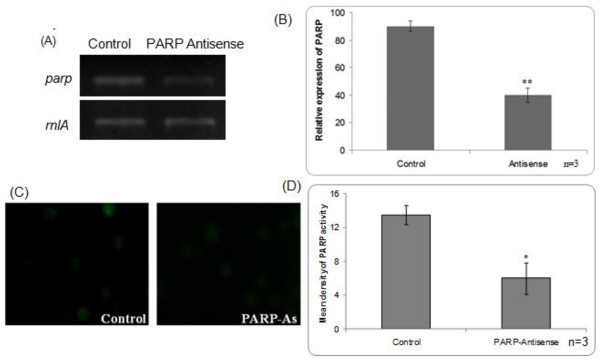
**Functional characterization of PARP antisense (As)**. (A) RT-PCR of PARP down-regulated *D. discoideum *cells. Gene specific expression of *parp *exhibits ~60% reduction while no change was observed with internal control *rnl*A. (B) Densitometric analysis of PARP expression by RT-PCR. (C) PARP activation monitored in PARP down-regulated *D. discoideum *cells by indirect immunofluorescence. PARP activity in down-regulated cells was found to be reduced compared to the control. (D) Densitometric analysis of PARP activity. ** p value <0.01; *p value <0.05 compared to control. The fluorescence photographs have been taken under 60X objective.

PARP down-regulation was confirmed by monitoring PARP expression by RT-PCR and it was found that PARP mRNA transcript was reduced by 60% (Figure 3A &3B). PARP activity was also monitored in these PARP down-regulated cells, and it was found to be lower than control cell basal activity (Figure 3C &3D). These results correlated well with the observed reduction in the PARP transcripts.

### Effect of PARP down-regulation on growth and development of *D. discoideum*

**Figure 4:**
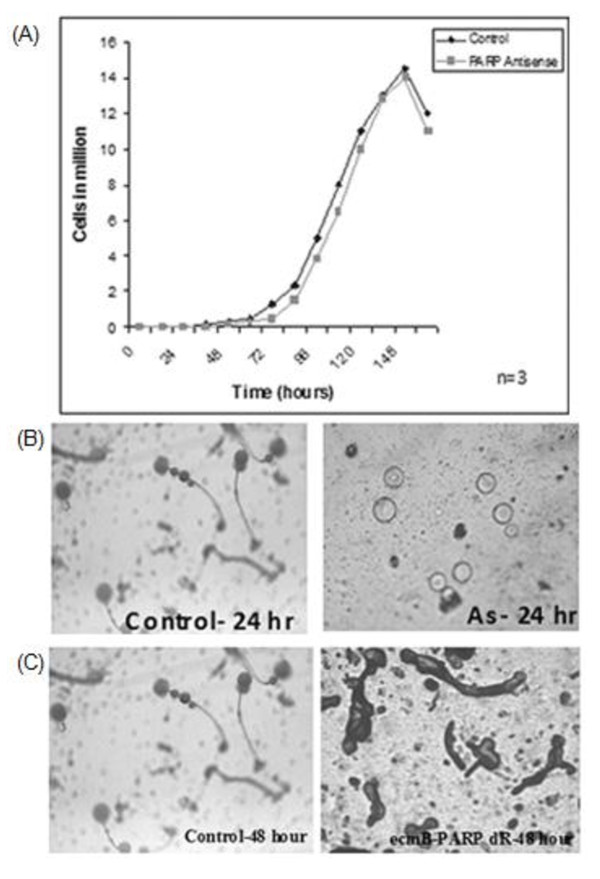
**Effect of PARP down-regulation on D. discoideum growth and development**. (A) Growth curve of PARP down-regulated *D. discoideum *cells. Growth in PARP down-regulated cells was comparable to control. Developmental arrest in *D. discoideum *cells with (B) constitutive and (C) stage specific down-regulation of PARP. The development was monitored after 6 hours and continued up to one week. The photographs have been taken under 4X objective.

PARP down-regulation did not show any effect on growth of the unicellular amoeba (Figure 4A) but interestingly when these cells were subjected to starvation induced development, morphogenesis was blocked at loose aggregation stage (Figure 4B). PARP down-regulated cells did not enter further development till one week. Moreover, stage specific PARP down-regulation in *D. discoideum *cells, arrested the development at slug stage (observed after 48 hours) signifying the involvement of PARP during development at different stages of differentiation (Figure 4C). Our results highlight the role of PARP in multicellularity as no effect was observed on growth of unicellular amoebae.

## Discussion

Oxidative stress induces delay or arrest of *D. discoideum *development [8]. The current study highlights the aggregative genes affected during oxidative stress and role of PARP during *D. discoideum *development. It was found that expression of *aca *and *car*1 were found to decrease with HA treatment (Figure 1). Benzamide did not show effect on expression of these genes (data not shown). This suggests that oxidative stress particularly affects certain pre-aggregation genes and decreased expression of these genes results in delayed development during oxidative stress.

### Role of PARP in *D. discoideum *development

The role of PARP is majorly established as NAD^+ ^dependent modifying enzyme that mediates important steps in DNA repair, transcription, and apoptosis, but its role during development is poorly understood. PARP deletion mutants in *Drosophila *fail to develop beyond larval stages due to defects in chromatin remodeling and regulation of gene expression [14]. In mouse PARP-1 and PARP-2 double knockouts exhibit embryonic lethality [15].

*D. discoideum *multiplies as a unicellular microorganism when food is abundant, but undergoes development on starvation; cells aggregate and differentiate, morphogenesis leads first to a migrating slug, then to a fruiting body with a mass of spores at the tip of a stalk composed of dead cells [16]. *D. discoideum *developmental cell death can occur in the absence of any member of the caspase family, making a constitutive link throughout evolution between this caspase family and programmed cell death unlikely. So far there are no reports which throw light on the protein/s involved in mediating *D. discoideum *paraptotic developmental cell death.

As the paraptotic cell death in unicellular stage of *D. discoideum *is found to be PARP mediated [17], we were interested to explore the involvement of PARP during its development also. Although oxidative stress did not affect the expression of *parp *but *parp *down-regulated cells when subjected to starvation interestingly failed to develop beyond loose aggregation stage (Figure 4B). PARP could be regulating certain key proteins of development. There could be two possibilities for the arrested development i.e., (1) PARP activity *per se *is required for transition from one stage to another and thus may directly influence the activity of protein/s required for development or (2) PARP may play a role in the regulation of developmental gene expression perhaps by "interacting with the promoters of these genes" or by poly ADP-ribosylation of certain transcription factors. Our results on prestalk stage specific down-regulation of PARP showed arrested development at the slug stage (Figure 4C). This accentuates that PARP induces stalk cell death in *D. discoideum *and thus opens up the possibility to further elucidate the role of PARP in its development.

Interestingly PARP is present in all multicellular organisms but not in the unicellular forms like yeast. This makes *D. discoideum *an excellent model system to study the role of PARP in development, as it is at the point of transition from unicellular to true multicelluar forms. Our results also suggest that presence of PARP in multicellular organisms may be linked to multicellularity. PARPs have been identified throughout the animal and plant kingdoms, with the catalytic domains exhibiting the greatest degree of sequence similarity. PARP is present in all types of eukaryotic cells with the notable exception of yeast, in which the expression of human PARP-1 was shown to lead to retarded cell growth [18]. A single PARP homolog (*prpA*) has also been reported in *Aspergillus nidulans *which is conserved in all filamentous fungi and is closely related to PARP-1. *Aspergillus nidulans *PARP ortholog (PrpA) revealed that the protein is essential in DNA repair, reminiscent of findings using mammalian systems. *A. nidulans *strain heterologous for *prp*A gene exhibited phenotypic defects in spore formation and possessed a pronounced fluffy phenotype caused by the inability to show asexual development [19]. Thus absence of PARP from unicellular organisms connotes its role in multicellularity.

In our studies PARP down-regulated *D. discoideum *cells get arrested at loose aggregation stage when subjected to development however, no effect was observed on the growth of unicellular *D. discoideum *(Figure 4A). These results support the idea that complex development and differentiation in filamentous fungi and *D. discoideum *may require additional programmed cell death pathways or components that are absent in yeast. Presence of PARP in *D. discoideum *and *A. nidulans *(filamentous fungi) signifies its role in multicellularity. However, further studies are needed to confirm the link between PARP and multicellularity.

## Conclusion

In the light of our results we propose that PARP plays an important role in multicellularity plausibly by regulating the developmental processes. *D. discoideum *being at the transition stage of multicellularity possesses PARP whereas unicellular fungi *S. cerevisaie *and *S. pombe *do not possess PARP. This new finding will undoubtedly influence our perception on PARP in developmental cell death in higher complex organisms including humans. Further work is to be done to explore the downstream targets of PARP during *D. discoideum *development.

## Authors' contributions

JR carried out most of the experiments and drafted the manuscript; HM performed RT-PCR for a few genes and helped in manuscript writing; RB participated in the design of the study and edited the manuscript. All authors have approved the final manuscript.
